# Treg regulation of the epithelial stem cell lineage

**DOI:** 10.1016/j.regen.2020.100028

**Published:** 2020-06

**Authors:** Inchul Cho, Prudence Pokwai Lui, Niwa Ali

**Affiliations:** a Centre for Stem Cells and Regenerative Medicine, School of Basic and Biomedical Sciences, King's College London, London, UK; b The Francis Crick Institute, London, UK

## Abstract

Tissue repair and maintenance in adult organisms is dependent on the interactions between stem cells (SCs) and constituent cells of their microenvironment, or niche. Accumulating evidence suggests that immune cells, specifically Foxp3^+^ CD4^+^ Regulatory T cells (Tregs), play an important role as a regulator of the SC niche. Undisputedly, Tregs are the major immunosuppressive lineage of the CD4^+^ T cell compartment, and reside within numerous secondary lymphoid organs, where they exert their functions. These cells are also specialised in facilitating protective functions specific to their tissue of residence. In this review, we discuss the emerging concepts supporting the SC-regulatory functions of tissue-resident Tregs, during both the steady-state and SC-mediated regeneration. We highlight the skin, intestines, and lung as model organs which are subject to recurrent microinjury,exposure to microbiota, and constantly replenished by resident stem cell populations. An in-depth understanding of the biology of the Treg-SC axis will inform ongoing immunotherapeutic endeavours to target specific subpopulations of tissue-resident Tregs.

## 1 Introduction

Successful tissue repair is largely dependent upon the efficient self-renewal and plasticity of stem cell (SC) populations that differentiate towards multiple cellular lineages. Some SC populations, including those present in the haematopoietic system, intestinal epithelium, or epidermis, constitutively regenerate tissue throughout the life-span of an organism. Long-term maintenance of a healthy tissue requires a finely tuned coordination between stem cells and constituent cells within the niche, while exempting exogenous threats, such as pathogens and toxins.

The epithelial barrier constitutes the first line of defence against external physical and chemical injury. The relationship between tissue maintenance and protection from external insults are well-illustrated during wound healing responses upon epithelial barrier breach. Initial injury invokes the recruitment and/or local activation of tissue-resident immune cells (TRICs) to sites of damage. This early immune response serves to protect the tissue against invading micro-organisms, and to clear damaged cells or cellular debris. The removal of damaged cells provides the spatial and signalling cue(s) necessary to induce epithelial SC proliferation and differentiation, thus replenishing the epithelium. The benefits and negative effects of immune inflammation on SC activation has been demonstrated by several previous studies, which have been reviewed.^1^^,^^2^ However, only a limited number of studies demonstrate direct immune cell regulation of epithelial SC activity.

Tissue-specific functions of immune cells, in particular, regulatory T cells (Tregs), have been documented in multiple non-lymphoid tissues, such as muscle and adipose tissue.^3^ Historically, research elucidating the existence of a direct immune cell-SC axis has been largely underexplored. This is despite the notion that TRIC activity is intimately associated with SC function, as observed during regenerative responses.^4^ Instead, the mechanisms influencing SC activity have been extensively studied in light of the surrounding epithelial cells and other stromal cells, which were largely driven by popularisation of the SC niche as a regulatory mechanism. The idea of the SC niche posits that SCs are regulated by cells and extracellular matrices directly within their microenvironment. Hence, immune cells, both resident and migratory in nature, have not been fully explored in this context.

Recent evidence suggests that multiple immune cell populations can directly interact with SCs to modulate their behaviour. Of which, Tregs are a prominent immune cell subset that reside in numerous peripheral tissues, where they are heavily implicated in SC regulation. In this review, we highlight recent evidence that supports the role of tissue-resident Tregs, not only as sentinels of the immune response, but as constituents of the epithelial SC niche. This is primarily exemplified in model organs that are subject to recurrent microinjury and exposure to microbiota, such as the intestines, lungs and the hair follicles (HFs) of skin.

## 2 The Treg lineage

Maintenance of healthy tissues requires the immune system to distinguish between self and non-self. In several organs, such as the skin, lungs and the intestines, where micro-organisms thrive, it is important to regulate over-active immune responses against self and commensal micro-organisms. As such, there are multiple distinct subsets of immunosuppressive TRICs, such as tolerogenic dendritic cells, innate lymphoid cells (ILCs), and Tregs. Their conventional immune functions have been previously reviewed.^5^, ^6^, ^7^

Regulatory T cells constitute a subset of CD4^+^ T cells that express the lineage defining transcription factor, forkhead box protein 3 (Foxp3). The majority of thymic Tregs (tTregs) develop in the thymus during thymocyte differentiation into mature T cells; whereas peripheral Tregs (pTregs) develop from naïve T cells in secondary lymphoid organs. The pTregs are then seeded into non-lymphoid organs, where they encounter tissue-specific antigens. Both within peripheral organs and in secondary lymphoid organs, Tregs suppress effector immune responses directed against self-antigens. This process is referred to as ‘immune tolerance’.

In humans, mutations in Foxp3 manifests in an autoimmune disease, termed immunodysregulation poly-endocrionopathy, X linked, or IPEX.^8^ The study of this disease, or the function of Foxp3-expressing Tregs, have been facilitated by the ‘scurfy’ mouse line, which harbour an insertional mutation at the Foxp3 locus. The resultant premature stop codon leads to the development of spontaneous autoimmunity.^9^ Follow up studies in mice further revealed specific expression of Foxp3 in CD4^+^ CD25^+^ peripheral Tregs,^10^ and that deletion of *Foxp3* phenocopies *Foxp3* mutant scurfy mice. It is now widely accepted that the high-affinity IL-2 receptor alpha chain, CD25 and the transcription factor Foxp3 are critical factors for Treg development, maintenance, and function.^10^, ^11^, ^12^

The delicate balance between immunosuppressive Tregs and effector T cells (Teffs) is crucial for the regulation of inflammation and self-tolerance. An imbalance in this dynamic, for instance, as a result of Treg ablation or dysregulation, underlies the pathology of numerous autoimmune diseases in both mice and humans.^13^ Moreover, genome-wide association studies (GWAS) have attributed autoimmune disease susceptibility in humans to impaired Treg function.^14^ Whilst functional Tregs are important for maintaining tissue homeostasis, the pathways they utilise may be hijacked by cancer cells to modulate the host immune system and dampen anti-tumour responses. As such, inhibition of Tregs and/or downstream pathways have emerged as promising candidates to promote anti-tumour immune responses.^15^ However, the current understanding of the diversity and molecular mechanisms that dictate tissue-specialised functions of resident Tregs remains incomplete. Establishing a solid foundational knowledge of the TRIC-SC axis will be crucial to fully elucidate the biology of auto-inflammatory and tissue-regenerative diseases.

## 3 Tregs and the skin stem cell niche

The skin constitutes the primary defence system against external insults and is the first organ to encounter pathogenic microbes. To facilitate defence against foreign bodies, the skin is equipped with a diverse range of active TRICs that communicate with local epithelia, ensuring robust immune responses upon the breach of the skin's barrier function. Amongst the numerous TRICs in murine skin, Tregs account for 20–60% of CD4^+^ T cells.^16^^,^^17^ Similarly, Tregs in human skin comprise 20% of total CD4^+^ T cells, as opposed to 5% in the peripheral circulation.^18^

It has recently been shown that an abrupt wave of Tregs are seeded into the skin early during postnatal development between postnatal day 6 to day 13, likely originating from the thymus.^17^ This Treg migratory event is facilitated by the expression of the chemokine CCL20 by the developing epithelial cells of the HF, attracting Tregs that express the chemokine receptor CCR6.^19^ Intriguingly, mice grown under germ-free conditions do not accumulate Tregs in this manner, suggesting that the process is dependent on the presence of microbiota, and that Tregs are involved in the regulation of immune homeostasis in the skin.^19^

Anatomically, the skin, or the epidermis, can be subdivided into the interfollicular epidermis (IFE), which forms the protective barrier, and the HFs, or the pilosebaceous unit, whose epithelium is contiguous with the IFE. The pilosebaceous unit is composed of distinct stem cell compartments, which can be identified by the expression of their respective molecular markers: Blimp1 (sebaceous gland stem cells); Gli1 and Lgr6 (lower isthmus stem cells); and integrin alpha-6 (ITGA6), Lgr5, CD34 and Krt15 (hair follicle stem cells (HFSCs)). It is speculated that these stem cells are restricted to their own lineage boundaries based on fate-mapping experiments.^20^, ^21^, ^22^, ^23^ The HFSCs are located in the lower portion of the epithelia, anatomically referred to as the ‘bulge’ region. These cells are responsible for mediating perpetual bouts of growth arrest (telogen) and activation (anagen) during the hair follicle (HF) cycle to form a new hair shaft.^24^ The HFSCs depend on signalling ligands, such as BMP, TGF-β and Wnt inhibitors to maintain quiescence^25^, ^26^, ^27^, ^28^; whereas the transition into a proliferative status requires activation of Wnt signalling.^28^^,^^29^ The differentiation trajectories of HFSCs are regulated by microenvironmental signals, which includes Notch signalling.^30^

Histological studies of human skin reveal that the majority of CD4^+^/Foxp3^+^ Tregs are preferentially associated with HFs, and rarely the interfollicular dermis or within the epidermis itself.^16^, ^17^, ^18^, ^19^^,^^31^ Flow cytometric analyses further revealed higher number of Tregs in the scalp and the face, which contain more HFs than other body sites. This is further supported by intravital multiphoton imaging of healthy mouse flank skin, showing bulge-associated Tregs displaying more dynamic activity than Tregs localised to other skin niches.^16^^,^^31^ Together, they present evidence that both human and murine skin Tregs are concentrated in the vicinity of follicular epithelium where HFSCs reside.^18^^,^^32^

The spatial proximity between HFSCs and Tregs suggest a possibility that the two cell types may engage in regulatory interactions. In line with this, human GWAS studies have linked single nucleotide polymorphisms in Treg-associated genes, including Foxp3, to alopecia areata, a disorder of immune-mediated disruption of HF regeneration.^33^^,^^34^ Importantly, augmenting Tregs clinically has shown efficacy in treating patients with this disease.^35^

**Fig. 1:**
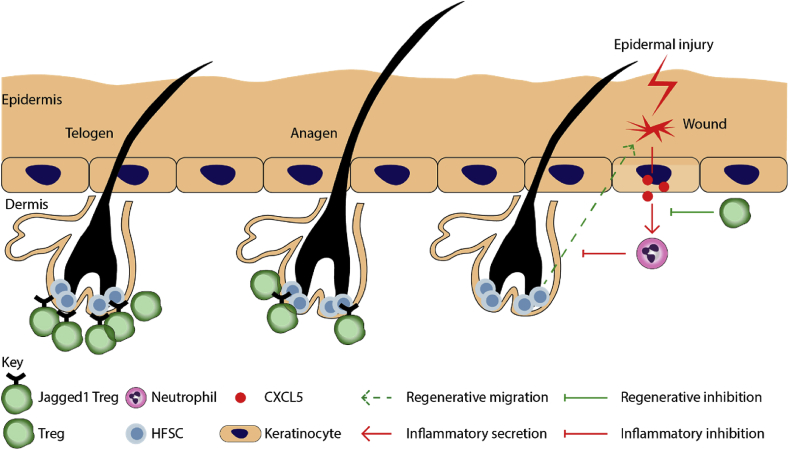
**Major functions of skin-resident Tregs in the regulation of epithelial SCs.** Tregs are highly abundant during the quiescent phase of the HF cycle, telogen, relative to the growth phase, anagen. Depletion of Tregs in Foxp3-DTR mice impairs HFSC activation and transition into anagen. A Treg-specific deletion of a notch ligand, Jagged1, phenocopies Treg depletion, suggesting that Tregs regulate HFSCs in a Jagged1-dependent manner. Upon epidermal injury, HFSCs are recruited to sites of wounding, where they contribute to epidermal regeneration. However, under inflammatory conditions, neutrophils inhibit the migration of HFSCs to sites of injury within the epidermis. Tregs facilitate the regenerative potential of HFSCs by inhibiting CXCL5-mediated activation of neutrophils, which would otherwise inhibit HFSC migration. Red arrows indicate inflammatory response. Green arrows indicate regenerative responses.

Curiously, the number of Foxp3^+^ Tregs closely associated with hair follicle cycle, declining during anagen, and increasing during , telogen (Fig. 1). Specific ablation of murine Tregs during telogen phase leads to impairment of the telogen-to-anagen transition, consequently reduces the proliferation and differentiation of CD34^+^ ITGA-6^+^ HFSCs, and impairs hair regeneration.^16^ Transcriptomic profiling further revealed a deficient Notch target gene expression profile in HFSCs in the absence of Tregs, and preferential expression of the Notch ligand, Jagged-1 (Jag1), in skin resident Tregs. Conditional deletion of Jag1 in Tregs using Foxp3^YFP−Cre^/Jag1^fl/fl^ mice largely phenocopied depletion of all skin Tregs, suggesting that the HFSC-regulatory role of Tregs is mediated via Jag1^16^. Given Tregs express a Notch ligand and HFSCs express Notch receptors suggest a possibility of direct cell-cell interaction. Supporting this notion, 2-photon intravital microscopy revealed that Tregs residing within 5 μm of the HF epithelium are more dynamically active, suggesting that a subpopulation of Tregs may directly interact with HFSCs.

After breach of the epithelial barrier upon full thickness wounding, Tregs rapidly accumulate in skin, and display a highly activated phenotype, as indicated by upregulation of Treg-associated activation markers - CD25^+^/CTLA-4^+^/ICOS^+^. Specific ablation of these cells in Foxp3-DTR mice via diphtheria toxin (DT) administration early post-wounding attenuates wound closure and re-epithelialization. The impairment of regeneration was attributed to an interferon-gamma dependent accumulation of Ly-6C^high^ pro-inflammatory macrophages (CD45^+^CD11b^high^F4/80^+^Ly-6C^high^Ly-6G^low^CD206^low^), in Treg-deficient mice.^36^ It was further discovered that infiltrating Tregs express the epidermal growth factor receptor (EGFR). Conditional deletion of EGFR in Tregs, using Foxp3^YFP−Cre^/EGFR^fl/fl^ mice, delayed wound closure and led to an increased accumulation of pro-inflammatory macrophages.^37^ These results suggest that the activation of Tregs upon injury may be dependent on EGF signalling. Cutaneous Tregs appear to co-opt a highly conserved regenerative pathway, often used by SCs as signals to enter the cell cycle, to mediate this specialised function of tissue repair. While these studies have demonstrated an active role for cutaneous Tregs during wound healing, whether Tregs directly modulate epithelial SC function, post full thickness wounding of skin, remains to be elucidated.

Indeed, a recent study has demonstrated an indirect role for Tregs in epidermal repair using *Foxp3-DTR* and Lgr5-TdTom mice. In this model, Tregs can be specifically ablated and Lgr5^+^ HFSCs simultaneously lineage-traced.^38^ Under conditions of epidermal injury as a result of repetitive tape stripping, injured epidermis secretes CXCL5, which causes an increase in neutrophilic infiltrate at sites of injury.^38^ Depletion of Tregs in this setting abrogates the regenerative impairment and is associated with neutrophilic infiltration and increased CXCL5. Recapitulating Treg-mediated repression of CXCL5 via neutralisation of CXCL5, partially rescued the impaired epidermal regeneration in Treg-depleted mice. These experiments indicate that Tregs control immune cell recruitment to regulate the inflammatory status of the skin. This, in turn, affects the ability of HFSCs to contribute to epidermal regeneration.^38^

These studies reveal that skin Tregs play a critical role in both HFSC and epithelial biology. Additionally, these findings provide a mechanistic link between tissue-resident Tregs and epithelial SCs that is necessary for adequate tissue function.

## 4 Tregs and the intestinal stem cell niche

In addition to the skin, the gut is under constant insult from chemicals and foreign microbes within the digestive tract. Hence, like the skin, the gut hosts a diverse repertoire of TRICs to ensure a robust response to injury. However, recurrent inflammation exposes the gut to an increased frequency of reactions directed against self-antigens. As such, the gut also hosts a large number of Tregs, perhaps in order to mitigate the anti-self response. In line with this, Treg numbers and functionality are significantly decreased in mouse models of inflammatory bowel disease.^39^^,^^40^ In human patients suffering from inflammatory bowel disease, circulating Treg numbers are decreased, whereas those that infiltrate the inflamed tissue increases. Moreover, reduction in circulating Treg numbers correlates with disease severity.^41^, ^42^, ^43^

Amongst murine intestinal resident CD4^+^ T cells, Tregs account for 25–35% in the colon.^44^ The number of colonic Tregs are significantly reduced in germ-free mice, suggesting their recruitment is dependent on chemokines that are secreted in the presence of microbiota.^45^ Numerous subsets of Tregs exist in the intestines that express transcription factors associated with T cell lineage differentiation, that in turn dictates the distinct nature of the immune response to be elicited.

**Fig. 2:**
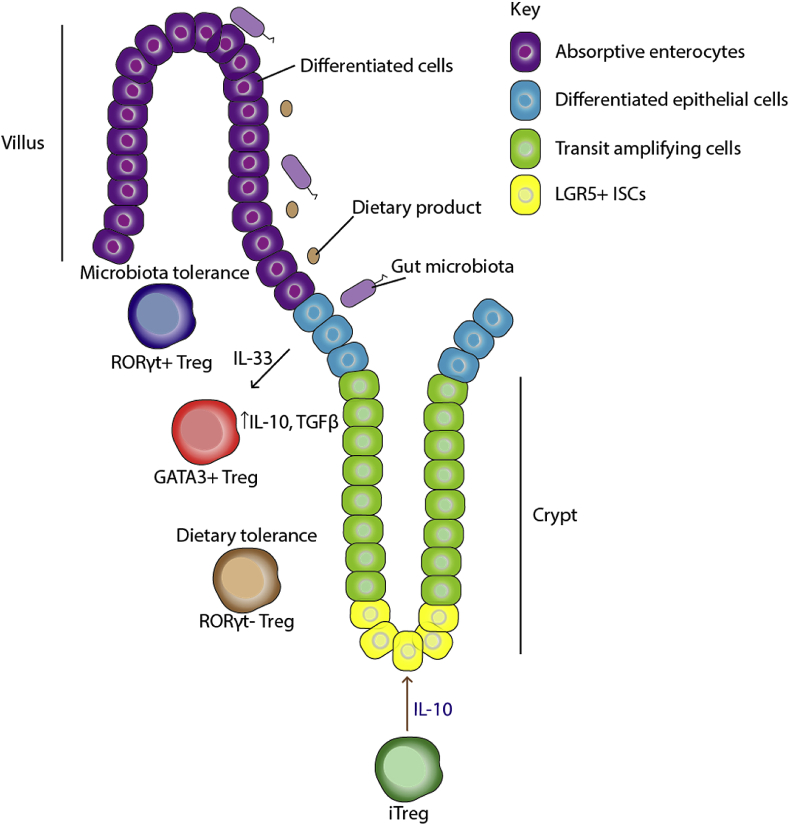
**Major functions of intestinal-resident Tregs in the regulation of epithelial SCs.** Several subpopulations of Tregs have been reported in the intestines with distinct roles in mediating tolerance towards dietary antigens and microbiota. Namely, RORγt- Tregs appear to be responsible for mediating dietary tolerance; RORγt + Tregs mediate microbial tolerance; whereas Gata3+ Tregs respond to the epithelial-derived interleukin-33, suggesting a possibility that they mediate epithelial-immune crosstalk. Indeed, IL-33 induces the production of anti-inflammatory cytokines TGFβ and IL-10 in Tregs. Finally, naïve T cells differentiated towards a Treg-like phenotype (iTreg, induced Tregs) by administration of TGFβ, support ISC growth *in vitro* by secretion of IL-10. Brown arrows indicate findings from *in vitro* studies.

Gut-resident Tregs can be subdivided into 1) GATA3^+^ Helios^+^ 2) RORγt^+^ and 3) RORγt^−^ Helios^−^ fractions (Fig. 2). The transcription factor GATA3 drives T helper 2 differentiation, but may also associate with the Foxp3 genomic locus, thus reinforcing Foxp3 expression to direct the Treg fate.^46^ Supporting this notion, Treg-specific deletion of GATA3 leads to destabilisation of the Treg phenotype under inflammatory conditions.^46^ Helios expression in Tregs was initially believed to definitively indicate thymic origin.^47^^,^^48^ However, this is challenged by a finding that the number of Helios^+^ Tregs increase in peripheral organs *in vivo* after stimulation with an antigenic peptide.^49^ Lastly, retinoic acid orphan receptor γt (RORγt) is the master regulator of Th17 cell differentiation.^50^ Overexpression of the RORC gene promotes expression of the main effector cytokine IL-17.^51^ Recent and emerging evidence suggests that Tregs acquire the lineage markers of T effector subsets that they regulate. For example, Tregs expressing the type 2 lineage marker, Gata3, preferentially regulate type 2 immune responses.^52^

The three subtypes of Tregs play distinct roles in the tissue micro-environment. GATA3^+^ Tregs have been shown to respond to interleukin-33 (IL-33) that is constitutively expressed by epithelial cells. Upon injury, IL-33 production is increased, which activates GATA3^+^ Tregs to further produce IL-10 and tumour growth factor-beta (TGF-β), both of which are anti-inflammatory.^53^ Whether these GATA3+ Tregs also express Helios, however, has not been studied. RORγt^+^ Tregs are responsible for tolerance to microbiota within the lumen.^54^ Tolerance is induced by the action of the transcription factor cMAF, which acts to dampen Th17 differentiation in colonic T cells.^55^ The last sub-type, RORγt^−^ Helios^−^ Tregs are less well defined functionally. Antibiotic treatment of mice does not affect their population size, whilst mice on antigen-free diets have reduced numbers of RORγt^−^ Helios^−^ Tregs, indicating a possible function in mediating tolerance against dietary antigens.^56^

Stem cell regulation mediated by Tregs has also been demonstrated in the intestines by a recent finding by Biton et al.^57^ The authors analysed intestinal epithelial cells via single cell RNA-sequencing (scRNA-seq) and identified three clusters of ISCs (ISC–I/II/III) that all express the SC marker, Lgr5. Amongst these clusters, ISC-1 cluster consisted of quiescent ISCs that expressed low levels of MHC-II, as opposed to the proliferative ISC-II/III clusters. The differences in MHC-II expression amongst these clusters suggest possible differences in their interactions with local immune cells. As such, the authors assessed possible interactions between ISCs and distinct T cell types using an *in vitro* organoid co-culture. In this setting, they observed an enhanced intestinal organoid renewal when co-cultured with induced-Tregs (T cells cultured *in vitro* in the presence of TGF-β) or IL-10, the main effector cytokine of Tregs. However, depletion of Tregs in Foxp3-DTR mice resulted not only in increased ISC proliferation, but alsodifferentiation into mature intestinal cells, such as tuft cells and goblet cells. The discrepancy between findings from *in vitro* and *in vivo* experiments may arise from several factors. Namely, the Tregs used for co-culture were derived from naïve T cells differentiated towards the Treg lineage via *in vitro* stimulation with TGF-β. In order to reconcile the different phenotypes, it will be necessary to fully characterise the cultured Tregs and determine which *in vivo* subtypes they most closely resemble. Moreover, there are several drawbacks of the method used in this study to quantify ISCs. The number of ISCs were indirectly assessed by quantification of Lgr5 transcripts, rather than total cell numbers. Taking this into account, the *in situ* immunofluorescence analyses did not reveal significant changes in the population size, or the number, of ISCs. Hence, a definitive functional role for Tregs in the direct modulation of ISC numbers remains an open question.

Other than Treg-mediated regulation of SC activity, Biton et al. demonstrated that local TRICS, other than Tregs, also possess the capacity to facilitate ISC differentiation. Specifically, antimicrobial Th1 responses direct ISC differentiation towards Paneth cells, which produce antimicrobial peptides. In contrast, the Th2 cells, which directs anti-parasitic responses, induces differentiation into tuft cells. These specialised cells possess the potential to differentiate into goblet cells, producing mucus directed to parasite eradication.^57^ Overall, these findings suggest an intriguing possibility that immune cells are able to utilise SC differentiation to enhance host defence. This was suggested by a previous study,^58^ which utilised ROR-⍺^sg/sg^ mice in which ROR⍺ is rendered non-functional via the introduction of a premature stop codon.^59^ In this model, type 2 innate lymphoid cells (ILC2) fail to develop under stimulatory conditions (in the presence of IL-25, a cytokine that expands and sustains ILC2s).^60^ Using this experimental approach, the authors assessed the importance of functional anti-parasitic ROR⍺^+^ ILC2s and their impact on mucus secreting goblet cells. The lack of functional ILC2s was associated with a marked decrease in the absolute number of goblet cells.^58^ These findings are consistent with previous studies that report a reduction in the level of type 2 cytokines in ROR⍺^sg/sg^ mice.^61^ Importantly, these findings shed light on the pathology of pathological conditions such as asthma, which has been associated with ROR⍺ mutations in a GWAS study.^62^ A recent *in vivo* finding demonstrated that upon infection, tuft cells constitutively express IL-25 to sustain and activate ILC2s, which subsequently secrete IL-13 to promote goblet cell differentiation.^60^ Given Foxp3 expression and Treg suppressive functions are enhanced by IL-25, it would be of interest to investigate whether Tregs are implicated in this immune-mediated differentiation.^63^

Overall, a direct or even indirect role, of Tregs in modulating ISCs is yet to be fully elucidated. An interesting avenue for further research would be to study how Tregs impact the classical signalling pathways that are known for the maintenance of the ISC niche. For instance, the activation of Wnt signalling pathway is sufficient to induce ISC proliferation,^64^^,^^65^ whereas genetic deletion of Wnt effector genes, such as β-catenin, results in loss the intestinal crypt.^66^ Intriguingly, it has recently been shown that Tregs express the membrane-bound Wnt antagonist, Dkk1.^67^ Whether tissue-resident Tregs also express Dkk1 is yet to be determined, however. Nonetheless, it raises an intriguing possibility that Dkk1-expressing Tregs, either resident or circulatory, may impact the Wnt signalling pathway in ISC clusters during homeostasis or injury-induced differentiation^68^

## 5 Tregs and the lung stem cell niche

As an integral part of the circulatory system, the most critical functions of the lungs are to expel respiratory waste products and to take in oxygen from the atmosphere. Consequently, the luminal surface of the lungs is constantly exposed to air-borne foreign particles. These include the influenza virus and particulate matters, which invoke immune cell responses. .Therefore, the lungs are also home to a diverse repertoire of TRICs.^69^^,^^70^

The lungs can be largely divided into three anatomically distinct segments; large airway, small airway, and the alveolus. Distinct SC lineages are known to exist along the epithelium, with the most multipotent basal cells populating the large airway, club cells lining the small airway, and unipotent type 2 alveolar epithelial cells (AEC2) in the alveolus, which differentiate into type 1 alveolar epithelial cells (AEC1s). The presence of bronchoalveolar stem cells have been demonstrated in the mouse bronchoalveolar duct junction, but not in humans.^71^

Perhaps owing to the presence of diverse SCs dedicated to maintaining their respective anatomical compartments, the lungs have remarkable regenerative capacity. Upon removal of one lobe of the lungs, the remaining lobes undergo compensatory growth to maintain the same level of respiratory volume.^72^ However, in end-stage lung diseases, such as chronic obstructive pulmonary disease and idiopathic pulmonary fibrosis (IPF), regenerative capacity is impaired. For instance, in IPF, AEC2s form bronchiolar-like structures rather than differentiating into AEC1s that line the alveolar epithelium.^73^

The pathological mechanisms of IPF are unclear. The current consensus posits that repeated insults overwhelm the regenerative potential of the AEC2s. In an effort to maintain barrier function, tissue-resident cells such as fibroblasts remodel the extracellular matrix irreversibly in IPF. Such changes within the microenvironment is likely to impact the behaviour of resident cells, including epithelial SCs, fibroblasts and immune cells, which remain incompletely understood.

The role of immune cells in the repair of lung epithelium upon injury has been previously demonstrated. For instance, depletion of tissue-resident ILC2s impaired regeneration post-influenza infection.^74^ Gene expression profiling of ILC2s revealed an abundant expression of amphiregulin, which alone was sufficient to restore barrier function in ILC2-depleted mice.^74^ Similarly, Tregs in the lungs have been shown to express amphiregulin, which was necessary for the re-establishment of barrier function in the lungs post-influenza infection.^75^ However, in both cases, whether barrier function was restored through extracellular matrix remodelling, or direct modulation of lung epithelial SCs has not been assessed. Nonetheless, these findings from mouse studies may be relevant to humans as amphiregulin-expressing Tregs have been found in peripheral organs, such as the lungs, where they also exert pro-regenerative functions.^76^

**Fig. 3:**
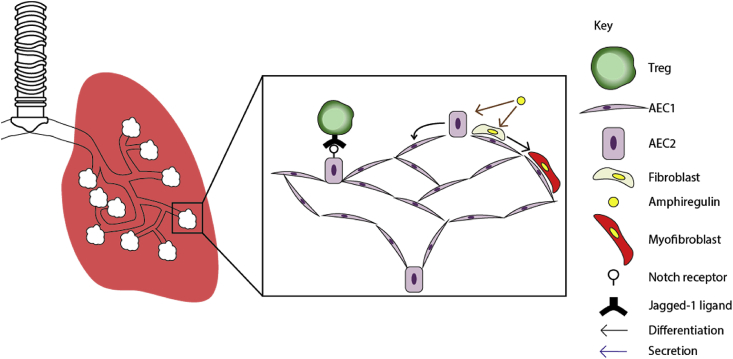
**Major functions of lung-resident Tregs in the regulation of epithelial SCs**. In the lungs, the alveolar epithelium is lined by AEC1s, which differentiate from AEC2s. The maintenance of AEC2s is partially regulated by Notch signalling activities. As such, it presents a possibility that Jagged1-expressing Tregs regulate AEC2 differentiation. A Treg-secreted molecule, amphiregulin, has been associated with restoration of barrier function in the lungs. However, whether Tregs are the primary source of amphiregulins in the lungs, and whether the functional recovery of the lungs were achieved as a result of epithelial stem cell proliferation, or deposition of extracellular matrix proteins, is incompletely addressed. Studies thus far suggest a dual role for amphiregulin. Amphiregulin has been shown to promote the differentiation of fibroblasts into myofibroblasts, which are specialised in remodelling the extracellular matrix. Additionally, recombinant amphiregulin protects AEC2s from apoptosis. Brown arrows indicate findings from *in vitro* studies.

It is currently unclear whether Tregs exert their regenerative functions in the lungs by acting on the stem cells, or the fibroblasts, to restore barrier functions in the lungs (Fig. 3). Additionally, the role of Tregs during degenerative processes, such as pulmonary fibrosis, remains controversial.^77^^,^^78^ Given that recurrent activation of fibroblasts underlie the pathology of idiopathic pulmonary fibrosis, it is of critical importance to fully characterise the target cells of Tregs, for an effective regenerative therapy that is without harmful side-effects, such as fibrosis.

Studies reported thus far indicate an essential role for immune cells during pulmonary repair, although the precise mechanisms by which the repair is mediated has not taken a direct TRIC-SC perspective. The role of amphiregulin has been discussed in this regard, which is secreted by Tregs and is critical for the recovery of barrier function. However, to our knowledge, a direct role for Tregs, or amphiregulin, in the induction of AEC proliferation is not established. There is also a lack of consensus on the role of amphiregulin in different modes of pulmonary injury.

## 6 Outlook for the Treg-stem cell axis in tissues

The widely held view that the dominant and sole function of the tissue immune system is protection against external insults is beginning to change. In particular, tissue-resident Foxp3^+^ Tregs possess numerous tissue-specific functions, outside of their conventional immunoregulatory role. In this review, we have discussed the SC-regulatory role of Tregs, evidence for which is beginning to accumulate. For instance, in the muscles, Tregs secrete the growth factor, amphiregulin to promote muscle satellite cell proliferation upon injury.^79^ Genetic ablation of Tregs has also been reported to result in impairment of pulmonary regeneration in multiple models of injury.^80^ We have also discussed SC-regulatory functions of Tregs in the skin, which is supported by conclusive evidence from Treg-specific deletion of a single factor in a model of SC-mediated regeneration. Jagged1-expressing Tregs facilitate HFSC proliferation and differentiation during the telogen-to-anagen transition of the HF cycle. The close proximity between skin Tregs and HFSCs would suggest either a direct interaction between Tregs and HFSCs or an indirect regulatory mechanism involving another proximal tissue cell type occurs. Indeed, the absence of Gata3+ Tregs in the skin leads to aberrant fibroblast activation and ECM remodelling, suggesting that there are additional mechanisms via which Tregs may regulate the HFSC microenvironment.^52^

Despite the presence of a diverse repertoire of TRICs in the lung, little is known of their influence on lung regeneration. Additionally, the lungs also harbour haematopoietic progenitors,^81^ inciting the notion that *de novo* generation of immune cells may occur to sustain immunological demands via processes that are regulated by TRICs, including Tregs. Crucially, we do not yet understand whether there are changes in processes such as haematopoietic activity and the interactions between TRICs and SCs upon injury, or in diseased states.

In light of pulmonary regeneration, it will be critical to understand the interactions between TRIC and SCs and the signalling mechanisms they employ. For example, resident alveolar macrophages (rAMs), a yolk-sac derived, self-renewing population of macrophages interacts with the airway epithelium. Cell-to-cell communication during the steady state and rAM-derived soluble mediators (e.g. CD200/R; IL-10, TGF-β, GMCSF) maintain homeostasis and quiescence.^82^ Loss of rAMs during injury may release these suppressive signals on AEC proliferation. In addition to these TRICs, others like CD103^+^ dendritic cells, intra-epithelial CD103^+^ memory T cells, interstitial macrophages (IMs), mucosa-associated invariant T cells (MAIT), invariant natural killer T cells (iNKT) cells and γẟ-T cells also reside either within the airway epithelium or in the mesenchyme surrounding the AEC. However, their contribution to AEC regeneration is unknown. Given that AEC2s rely on Notch signalling for differentiation into AEC1s,^83^ it is interesting to speculate that Jag1-expressing Tregs may be involved in alveolar differentiation. Currently, a role for alveolar Tregs in AEC2 maintenance or proliferation post-injury has not been identified. Given the recent demonstration that a distinct subset of cutaneous Tregs are poised for the regulation of fibroblasts, and therefore, fibrosis,^52^ it may be functionally relevant to fully characterise alveolar resident Tregs. It is possible a subset of Tregs in this location can influence fibroblast behaviour, and/or epithelial SC homeostasis, in a manner similar to that of skin. Such identifications will allow for subsequent therapies that ameliorate fibrosis.

To date, regenerative functions of tissue-resident Tregs have largely been explored in light of amphiregulin secretion and its downstream targets.^75^^,^^79^^,^^84^^,^^85^ However, tissue-specific molecular signatures of Tregs, other than amphiregulin, has been suggested to play a critical role in mediating regeneration of the spinal cord, heart and retina of zebrafish.^86^ Indeed, recent reports form studies of mouse skin suggest that Tregs can regulate regeneration via additional mechanisms, such as Notch signalling pathways, and modulating the activation of other infiltrating immune cells.^16^^,^^38^ As such, an interesting avenue for further research would be to fully characterise the diversity of Tregs and their tissue-specific molecular signatures that are vital for the homeostasis of epithelial SCs within individual organs.

Why are immunosuppressive Tregs so intimately associated with the regulation of SC function? One line of relevant evidence demonstrates that quiescent SCs, such as those that reside in HFs, downregulate the expression of MHC-I to evade the immune system. In contrast, fast-cycling SCs begin to express MHC-I and are thereby recognised and targeted for immune-mediated clearance.^87^ This appears paradoxical as an immune-mediated attack on SCs would compromise the ability of SCs to re-populate the damaged epithelium. One possibility is that Tregs suppress over-activated immune cell subsets that secrete or promote factors that negatively perturb SC as they enter the cycle. Intriguingly, Treg suppressive phenotypes are reinforced by receiving EGF signalling, such as amphiregulin.^85^ This suggests a requirement for a local immunosuppressive environment during the post-injury tissue regenerative phase.

The SC-Treg axis sheds an important and new light on cancer immunology. In this context, Tregs are viewed as suppressors of the immune system that nullify host defence mechanisms.^88^ Given the many parallels between the mechanisms by which cancer and SCs are regulated, it is possible that tumour initiating, or progenitor-like SCs, may interact with Tregs in manners similar to that reviewed here, which will be a clinically relevant avenue for further research.

The collective view indicates that Tregs mediate the majority of their functions in their respective tissues of residence. It is therefore likely that strategies targeting the augmentation or suppression of specific Treg subsets will result in optimal therapeutic outcomes. Ultimately, a fundamental understanding of how Tregs impact tissue regeneration, via modulation of SC activity, will be of critical importance in the maintenance and restoration of tissue homeostasis.
